# Comparing antibiotic prescribing patterns for hidradenitis suppurativa between dermatology and non‐dermatology ambulatory providers

**DOI:** 10.1002/ski2.451

**Published:** 2024-08-28

**Authors:** Hannah Tolson, Robin Kikuchi, Rebecca K. Yamamoto, Kaviyon Sadrolashrafi, Lily Guo, Audrey Hao, Sara Bilimoria, Danielle Yee, April W. Armstrong

**Affiliations:** ^1^ University of Arizona College of Medicine Phoenix Arizona USA; ^2^ Keck School of Medicine of University of Southern California Los Angeles California USA; ^3^ Georgetown University School of Medicine Washington District of Columbia USA; ^4^ Kirk Kerkorian School of Medicine at UNLV Las Vegas Nevada USA; ^5^ Duke University School of Medicine Durham North Carolina USA; ^6^ Division of Dermatology Department of Medicine David Geffen School of Medicine at the University of California Los Angeles California USA

## Abstract

**Background:**

Oral antibiotics are a mainstay of treatment for hidradenitis suppurativa (HS) primarily due to their anti‐inflammatory and anti‐microbial properties. Because antibiotics are frequently prescribed to treat HS, concerns exist regarding antibiotic stewardship. There is a paucity of literature comparing how antibiotic prescribing patterns for HS differ between dermatology and non‐dermatology clinicians in the ambulatory setting.

**Objective:**

This study aims to compare the antibiotic prescribing patterns of dermatology versus non‐dermatology clinicians treating HS in the ambulatory setting.

**Methods:**

We utilised the National Ambulatory Medical Care Survey (NAMCS) to identify visits for HS patients from 2005 to 2016. We performed multivariate logistic regression analysis to compare the likelihood of prescribing (1) antibiotics and (2) tetracyclines between dermatology and non‐dermatology clinicians in the ambulatory setting.

**Results:**

We identified a total of 2 424 125 (weighted) HS visits. Approximately 28.0% of visits were conducted by dermatology clinicians, while 72.0% were conducted by non‐dermatology clinicians. Antibiotics were prescribed in 51.9% of visits. Tetracyclines were the most commonly prescribed antibiotics among visits with dermatology clinicians (33.4%), while penicillins/cephalosporins were the most commonly prescribed antibiotic among visits with non‐dermatology clinicians (14.9%). Multivariate logistic regression analysis demonstrated no difference in the overall likelihood of prescribing antibiotic therapy between dermatology and non‐dermatology clinicians (*p* = 0.35). However, dermatology clinicians were significantly more likely to prescribe tetracyclines than non‐dermatology clinicians (OR 5.48, 95% CI 1.19–25.26, *p* = 0.03).

**Conclusion:**

In conclusion, dermatology clinicians were significantly more likely to prescribe tetracyclines than non‐dermatology clinicians for HS patient visits.

## Graphical abstract

Oral antibiotics are a mainstay of treatment for hidradenitis suppurativa (HS), and HS is treated by dermatology and non‐dermatology clinicians. This study aims to compare the antibiotic prescribing patterns of dermatology versus non‐dermatology clinicians treating HS in the ambulatory setting. In this study, dermatology clinicians were significantly more likely to prescribe tetracyclines than non‐dermatology clinicians for HS patient visits.

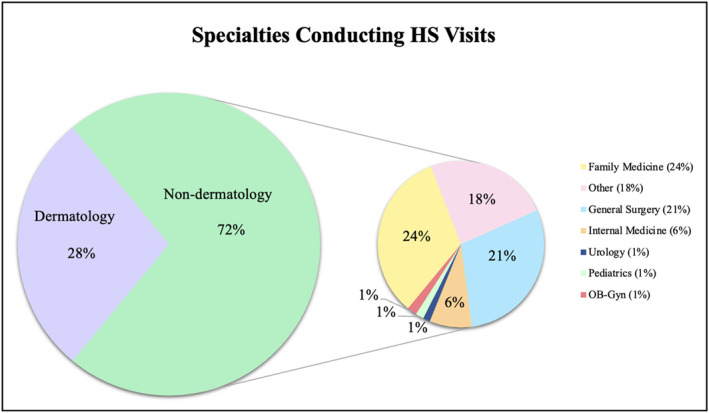

**What is already known?**

- Historically dermatologists prescribe more oral antibiotics per clinician than any other speciality. Oral antibiotics are a mainstay of treatment for hidradenitis suppurativa (HS), an autoimmune skin disease managed by dermatology and non‐dermatology clinicians.

**What does this study add?**

- Our study found that dermatology clinicians were more likely to prescribe tetracyclines for HS than non‐dermatology clinicians. These findings suggest that prescribing patterns among dermatology clinicians may follow antibiotic treatment guidelines more closely, which recommend tetracyclines as first‐line therapy.

## 1 INTRODUCTION

Hidradenitis suppurativa (HS) is a chronic, inflammatory skin disease that presents with painful nodules and abscesses in intertriginous areas.^1^ HS is poorly understood and difficult to manage, with treatment options including topical therapies, antimicrobials, hormonal therapies, and a wide range of immunomodulating medications.^2^ Despite new advances in therapy such as biologics, oral antibiotics remain a cornerstone of treatment for HS.^3^

Because antibiotics are frequently prescribed to treat HS, concerns exist regarding antibiotic stewardship.^4^ Patients with HS demonstrate high rates of antibiotic resistance and reduced sensitivity to standard antibiotic regimens.^5^ Evidence‐based guidelines exist to reduce the development of antibiotic resistance and ensure maximal therapeutic benefit. These guidelines outline oral tetracyclines as a first‐line choice due to their superior performance in randomized controlled trials.^2^

Patients with HS are cared for by clinicians (physicians, nurse practitioners, and physician assistants) whose specialities range from dermatology to general surgery, obstetrics/gynaecology (ob‐gyn) and family medicine. Often, HS is managed in the ambulatory setting, also referred to as the outpatient (or non‐hospital) setting. On the basis of speciality training, clinicians may have different levels of education and experience in treating dermatologic diseases such as HS, particularly in the context of antibiotic stewardship.^4^ As the treatment landscape for HS evolves, a notable gap has emerged in the literature regarding antibiotic prescribing patterns among different medical specialities. This study aims to compare the likelihood of prescribing (1) any antibiotics and (2) tetracyclines among dermatology versus non‐dermatology clinicians in the ambulatory setting.

## 2 METHODS

We identified visits for patients with HS from 2005 to 2016 from the National Ambulatory Medical Care Survey (NAMCS). The NAMCS estimates a nationally representative sample of patient visits using a complex probability survey design with masked weighting variables. Variable weighting is recommended to ensure accurate data analysis.^6^ Information for NAMCS was collected for practice and physician characteristics.^7^ As all data collected in this study were de‐identified, this study was considered exempt by the University of California, Los Angeles Institutional Review Board.

We identified visits for patients with HS using the *International Classification of Diseases, Ninth Revision* (*ICD‐9*) code 705.83 and the *International Classification of Diseases, Tenth Revision* (*ICD‐10*) code L73.2 between 1 January 2006 and 31 December 2016.

The variable of interest was the prescription of oral antibiotics indicated by medications from any of the following classes: tetracyclines, clindamycin, penicillins/cephalosporins, fluoroquinolones, dapsone, rifampin, and TMP/SMX. These antibiotics have been identified in the literature as commonly prescribed antibiotics for the management of HS.^8^ Patients prescribed multiple antibiotics were counted only once as receiving antibiotics. We also obtained information regarding the number of biologic therapies prescribed to use as a reference.

The independent variable was the ambulatory speciality denoted as dermatology or non‐dermatology. We classified the following specialities as non‐dermatology: family medicine, internal medicine, general surgery, ob‐gyn, urology, paediatrics and ‘other’ specialities, which was a designation given to physicians whose specialities were not listed in the database.

We calculated descriptive statistics for patient demographics, clinical characteristics and patient outcomes. Continuous variables were reported with mean and standard deviation. Categorical variables were reported with (weighted) raw numbers and proportions. We performed frequency counts for antibiotic prescriptions for the total population and the population stratified by speciality (dermatology vs. non‐dermatology). Multivariate logistic regression analysis was performed using (1) antibiotic prescriptions and (2) tetracycline prescriptions as the outcome variable and speciality (dermatology vs. non‐dermatology) as the independent variable. The logistic regression models were adjusted for age, sex, insurance type, race/ethnicity, medical comorbidities (measured by the Charlson Comorbidity Index) and rural/urban status. We defined the significance threshold as a *p*‐value less than 0.05. All data management and analysis tasks were conducted using Stata 18.0 statistical software.

## 3 RESULTS

**FIGURE 1:**
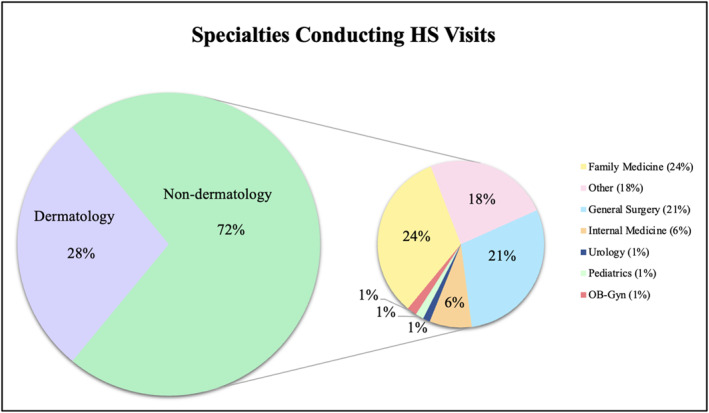
Specialties conducting HS patient visits. Non‐dermatology is further stratified as family medicine, general surgery, internal medicine, urology, paediatrics, ob‐gyn, and other.

We identified a total of 2 424 125 (weighted) HS visits. Approximately 28.0% were conducted by dermatology clinicians while 72.0% were conducted by non‐dermatology clinicians. The three non‐dermatology specialities that conducted the most HS visits were family medicine (24%), general surgery (21%) and ‘other’ (18%), which was a designation utilised by the database for all specialities that were not identified. The complete sociodemographic data for visits is presented in Table 1. Speciality proportions are displayed in Figure 1.

**TABLE 1:** Sociodemographic characteristics of HS patient visits from 2005 to 2016 in the NAMCS database.

Characteristic	Overall visits Weighted *n* = 2 424 125	Dermatology visits Weighted *n*, (%) = 678 576 (28.0)	Non‐dermatology visits Weighted *n*, (%) = 1 745 549 (72.0)	*p*‐value
Antibiotics, *n* (%)				
Prescribed	1 165 189 (51.9)	389 068 (57.3)	776 122 (44.4)	0.01^b^
Not prescribed	1 258 936 (48.1)	289 508 (42.6)	969 427 (55.6)	
Sex, *n* (%)				
Male sex	685 335 (28.3)	140 308 (20.7)	545 027 (31.2)	0.66^b^
Female sex	1 738 790 (71.7)	538 268 (79.3)	1 200 522 (68.8)	
Age, mean (SEM) years	37.2 (1.8)	36.5 (2.1)	37.6.8 (2.3)	0.57^a^
Insurance, *n* (%)				
Private	1 396 558 (57.6)	429 344 (63.3)	967 214 (55.4)	0.43^b^
Medicare	224 717 (9.3)	110 081 (16.2)	114 636 (6.6)	
Self‐pay	38 268 (1.6)	9464 (1.4)	28 804 (1.6)	
Medicaid or CHIP	651 968 (26.9)	128 966 (19.0)	523 002 (30.0)	
Other unknown	112 614 (4.6)	721 (0.1)	111 893 (6.4)	
Race/Ethnicity, *n* (%)				
White only	1 580 236 (65.2)	495 118 (73.0)	1 085 118 (62.2)	0.01^b^
Black only	641 718 (26.5)	54 683 (8.1)	587 035 (33.6)	
Hispanic	169 413 (7.0)	117 627 (17.3)	51 885 (3.0)	
Other race/Multiple race	32 758 (1.3)	11 248 (1.6)	21 511 (1.2)	
CCI, mean (SEM)	0.4 (0.1)	0.5 (0.1)	0.35 (0.1)	0.42^a^
MSA status, *n* (%)				
Urban	2 256 900 (93.1)	678 576 (100.0)	1 578 324 (90.4)	0.05^b^
Rural	157 225 (6.9)	• (0.0)	167 225 (9.6)	

^a^ Analysis of variance of the differences among visits with HS patients of different ages and Charlson Comorbidity Indices (CCI).

^b^ *X* ^2^ test of the differences among visits with HS patients of different race/ethnicity, insurance, rural/urban status, and sex.

**FIGURE 2:**
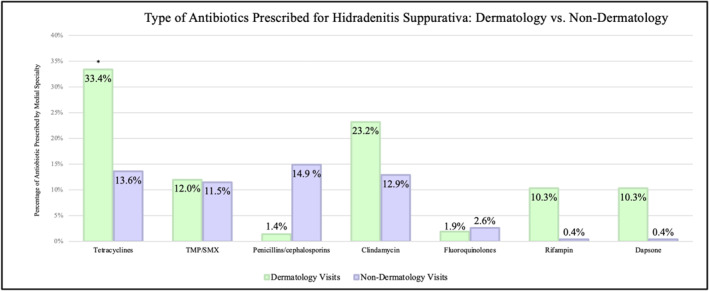
Frequency of each antibiotic prescribed stratified by specialty (dermatology vs. non‐dermatology). *indicates a *p*‐value <0.05 in chi‐squared testing between the two specialties.

Overall, 51.9% of all HS visits resulted in antibiotic prescriptions. Approximately 57.3% of dermatology visits resulted in oral antibiotic prescriptions and 44.4% of non‐dermatology visits resulted in antibiotic prescriptions. Tetracyclines were the most commonly prescribed antibiotic among dermatology clinicians (33.4% of visits) while penicillins/cephalosporins were the most commonly prescribed antibiotics among non‐dermatology clinicians (13.6%). Complete chi‐squared results are presented in Table 2 and Figure 2.

**TABLE 2:** Medications prescribed in HS patient visits according to speciality between 2005 and 2016 in the NAMCS database.

Antibiotics prescribed	Overall visits Weighted *n* = 2 424 125	Dermatology visits Weighted *n* = 678 576	Non‐dermatology visits Weighted *n* = 1 745 549	*p*‐value
Tetracyclines, *n* (%)	464 599 (19.2)	226 981 (33.4)	237 618 (13.6)	<0.0001^a^
TMP/SMX, *n* (%)	281 441 (11.6)	81 469 (12.0)	199 972 (11.5)	0.39^a^
Penicillins/cephalosporins, *n* (%)	269 232 (11.1)	9464 (1.4)	259 767 (14.9)	0.18^a^
Clindamycin, *n* (%)	382 536 (15.8)	157 629 (23.2)	224 907 (12.9)	0.01^a^
Fluoroquinolones, *n* (%)	57 649 (2.4)	13 026 (1.9)	44 623 (2.6)	0.85^a^
Rifampin, *n* (%)	77 339 (3.2)	69 905 (10.3)	7434 (0.4)	0.14^a^
Dapsone, *n* (%)	77 339 (3.2)	69 905 (10.3)	7434 (0.4)	0.14^a^
Humira, *n* (%)	5168 (0.2)	5168 (0.8)	‐	‐

^a^ *X* ^2^ test of the differences in the frequency of antibiotic classes prescribed for HS patient visits (between dermatologists and non‐dermatologists).

We performed multivariate logistic regression analysis to compare the likelihood of prescribing (1) antibiotics and (2) tetracyclines between dermatology and non‐dermatology clinicians. We found no difference in the odds of prescribing antibiotic therapy between dermatology and non‐dermatology clinicians (*p* = 0.35). However, dermatology clinicians were significantly more likely to prescribe tetracyclines than non‐dermatology clinicians (OR 5.48, 95% CI 1.19–25.26, *p* = 0.03). Multivariate logistic regression results are presented in Tables 3 and 4.

**TABLE 3:** Multivariate logistic regression analysis of the association between antibiotic prescriptions and medical speciality in HS patient visits, adjusting for sex, race/ethnicity, insurance type, age, rural/urban status and Charlson comorbidity index.

Antibiotics versus no antibiotics	OR (95% CI)	*p*‐value
Visit type		
Ambulatory non‐dermatology	(Ref)	‐
Ambulatory dermatology	1.88 (0.49–7.21)	0.35
Sex		
Female	(Ref)	‐
Male	0.32 (0.07–1.45)	0.13
Age	1.03 (0.97–1.1)	0.34
Insurance type		
Private insurance	(Ref)	‐
Medicare	0.61 (0.07–5.47)	0.65
Self‐pay	3.18 (0.31–33.04)	0.32
Medicaid	1.28 (0.22–7.31)	0.78
Other	0.41 (0.28–5.85)	0.50
Race		
White	(Ref)	‐
Black	1.61 (0.41–6.28)	0.87
Hispanic	0.51 (0.09–3.01)	0.44
Other race	1.14 (0.11–11.02)	0.91
Charlson comorbidity index	0.62 (0.20–1.94)	0.40
Rural/Urban status		
Urban	(Ref)	‐
Rural	0.16 (0.01–2.97)	0.21
F(12, 19)	0.54	
Prob > F	0.86	

**TABLE 4:** Multivariate logistic regression analysis of the association between tetracycline prescriptions and medical speciality in HS patient visits, adjusting for sex, race/ethnicity, insurance type, age, rural/urban status and Charlson comorbidity index.

Tetracyclines versus no tetracyclines	OR (95% CI)	*p*‐value
Visit type		
Ambulatory non‐dermatology	(Ref)	‐
Ambulatory dermatology	5.48 (1.19–25.26)	0.03
Sex		
Female	(Ref)	‐
Male	0.13 (0.02–0.72)	0.02
Age	1.08 (0.99–1.18)	0.08
Insurance type		
Private insurance	(Ref)	‐
Medicare	0.15 (0.02–1.15)	0.07
Self‐pay	6.62 (0.55–80.26)	0.13
Medicaid	0.24 (0.02–2.80)	0.24
Other	2.27 (0.13–38.98)	0.56
Race		
White	(Ref)	‐
Black	0.82 (0.20–3.42)	0.78
Hispanic	0.34 (0.05–2.32)	0.26
Other race	3.60 (0.74–17.44)	0.11
Charlson comorbidity index	0.52 (0.14–2.01)	0.33
Rural/Urban status		
Urban	(Ref)	‐
Rural	1.77 (0.07–46.17)	0.72
F(12, 19)	1.12	
Prob > F	0.40	

## 4 DISCUSSION

This cross‐sectional study characterises the antibiotic prescribing patterns of dermatology and non‐dermatology clinicians treating HS. Antibiotics were prescribed in 57.3% of dermatology visits and 44.4% of non‐dermatology visits. We found no difference in the likelihood of prescribing antibiotics between dermatology and non‐dermatology clinicians. However, dermatology clinicians were more likely to prescribe tetracyclines than non‐dermatology clinicians (Tables 3 and 4).

Historically, dermatologists prescribe more oral antibiotics per clinician than any other speciality.^9^ However, recent trends demonstrate a decline in antibiotic use among clinicians practicing dermatology.^10^ One explanation for this is increased awareness of antibiotic stewardship and improved strategies for managing chronic skin conditions, including the use of biologics.^11^ In our study population, biologics were prescribed during 5168 HS visits and were exclusively prescribed by dermatology clinicians. Additionally, among patients who are prescribed biologics, none were concurrently prescribed oral antibiotics. Our results may indicate that dermatologists preferentially opt for biologic and other non‐antibiotic therapies. As a result, the difference in antibiotic prescribing rates between dermatology and non‐dermatology clinicians may be narrowing, leading to the negligible discrepancy that we observed.

Although there is no significant difference in antibiotic‐prescribing rates between dermatology and non‐dermatology clinicians, we found that dermatology clinicians were more likely to prescribe tetracyclines to their HS patients (Table 4). We also noted similarities in the frequency of prescriptions written by non‐dermatology clinicians within antibiotic classes: tetracyclines (13.6%), TMP/SMX (11.5%), beta‐lactams (14.9%) and clindamycin (12.9%) (Figure 2). Oral tetracyclines have been shown in some studies to be among the first‐line choices due to their superior performance in randomized controlled trials.^2^ This is likely due in part to the anti‐inflammatory effects that tetracyclines exhibit. For this reason, national and international guidelines for the treatment of mild‐to‐moderate HS recommend using only tetracyclines as oral antibiotic monotherapy.^2^, ^12^ However, these guidelines are typically utilised within the dermatology community and may not readily reach other clinicians.^2^ Therefore, our results may be explained by a lack of access to treatment guidelines for non‐dermatology clinicians, and consequently, less familiarity with treating the disease.

The findings of this study should be interpreted in the context of the study design. The severity of the disease of individuals' cannot be ascertained in most large database studies. While it would be preferable to utilise the severity of HS in our analysis, as severity often dictates treatment choice, NAMCS does not capture severity using validated measures.

While the therapeutic armamentarium of HS has evolved substantially in the last decade, antibiotics continue to play a fundamental role in multi‐modal treatment.^4^, ^13^, ^14^, ^15^ Clinicians must carefully consider their choice of antibiotic class and dosing frequency to avoid resistance. Approximately 72.0% of HS patient visits were conducted by non‐dermatology clinicians, meaning that familiarity with management is imperative across a broad spectrum of medical specialities. Furthermore, attention should be paid to specialists using antibiotics for HS in the acute setting, such as general surgeons who prescribe antibiotics perioperatively. Future work may be directed towards studying biologic prescribing patterns between dermatology and non‐dermatology clinicians treating HS to better characterise barriers to appropriate long‐term management for HS patients.

## 5 CONCLUSION

In conclusion, dermatology and non‐dermatology clinicians demonstrated a significant difference in tetracycline prescribing rates. These findings suggest that prescribing patterns among dermatology clinicians may follow antibiotic treatment guidelines more closely, which recommend tetracyclines as a first‐line therapy. As both dermatology and non‐dermatology clinicians manage HS, efforts should be made to ensure that proper exposure and education are provided to all physician specialities.

## CONFLICT OF INTEREST STATEMENT

AWA has served as a research investigator, scientific advisor and/or speaker to AbbVie, Almirall, Arcutis, ASLAN, Beiersdorf, BI, BMS, EPI, Incyte, Leo, UCB, Janssen, Lilly, Mindera, Nimbus, Novartis, Ortho Dermatologics, Sun, Dermavant, Dermira, Sanofi, Regeneron and Pfizer. All other authors have no conflicts to disclose.

## AUTHOR CONTRIBUTIONS

**Hannah Tolson**: Conceptualisation (lead); Data curation (lead); Formal analysis (lead); Writing—original draft (lead). **Robin Kikuchi**: Conceptualisation (equal); Writing—review & editing (equal). **Rebecca K. Yamamoto**: Formal analysis (equal); Writing—original draft (equal); Writing—review & editing (equal). **Kaviyon Sadrolashrafi**: Writing—original draft (equal); Writing—review & editing (equal). **Lily Guo**: Writing—original draft (equal); Writing—review & editing (equal). **Audrey Hao**: Writing—original draft (equal); Writing—review & editing (equal). **Sara Bilimoria**: Writing—review & editing (equal). **Danielle Yee**: Writing—review & editing (equal). **April W. Armstrong**: Methodology (equal); Writing—review & editing (equal).

## ETHICS STATEMENT

University of Californa, Los Angeles IRB#23–001697. Based on the information provided in the webIRB application, the UCLA Office of the Human Research Protection Programme has determined that the above‐named project does not meet the definition of human subject research.

## PATIENT CONSENT

Not applicable.

## Data Availability

The data underlying this article will be shared with the corresponding author on reasonable request.
